# Synthesis and Characterization of Core-Shell Acrylate Based Latex and Study of Its Reactive Blends

**DOI:** 10.3390/ijms9030342

**Published:** 2008-03-12

**Authors:** Xiang Liu, Xiao-Dong Fan, Min-Feng Tang, Ying Nie

**Affiliations:** 1 Department of Applied Chemistry, School of Science, Northwestern Polytechnical University Xi’an 710072, P. R. China; E-mails: lxhx@xsyu.edu.cn; xfand@126.com; nputmf@126.com; 2 School of Chemistry and Chemical Engineering, ShiYou University, Xi’an 710065, P. R. China; E-mail: nieying@126.com

**Keywords:** core-shell morphology, latex/resin blends, waterborne melamine-formaldehyde resin, waterborne urea-formaldehyde resin

## Abstract

Techniques in resin blending are simple and efficient method for improving the properties of polymers, and have been used widely in polymer modification field. However, polymer latex blends such as the combination of latexes, especially the latexes with water-soluble polymers, were rarely reported. Here, we report a core-shell composite latex synthesized using methyl methacrylate (MMA), butyl acrylate (BA), 2-ethylhexyl acrylate (EHA) and glycidyl methacrylate (GMA) as monomers and ammonium persulfate and sodium bisulfite redox system as the initiator. Two stages seeded semi-continuous emulsion polymerization were employed for constructing a core-shell structure with P(MMA-*co*-BA) component as the core and P(EHA-*co*-GMA) component as the shell. Results of Transmission Electron Microscopy (TEM) and Dynamics Light Scattering (DLS) tests confirmed that the particles obtained are indeed possessing a desired core-shell structural character. Stable reactive latex blends were prepared by adding the latex with waterborne melamine-formaldehyde resin (MF) or urea-formaldehyde resin (UF). It was found that the glass transition temperature, the mechanical strength and the hygroscopic property of films cast from the latex blends present marked enhancements under higher thermal treatment temperature. It was revealed that the physical properties of chemically reactive latexes with core-shell structure could be altered via the change of crosslinking density both from the addition of crosslinkers and the thermal treatment.

## 1. Introduction

Since the idea of “particle design” was put forward by Okubo in 1980s [1–5], where the control of micro-phase separated structure of latex particles via controlling the distribution of hetero-functional groups in their internal particles or on their surface were the main focus, various latex particles with different morphology, such as double or multi-layers structures, were obtained via specially macromolecular designing and emulsion polymerization techniques. It was found that the composite latexes were also possessing distinguished physical properties [6–8]. Therefore, the “particle design” as a novel technology in the field of micro-composite science attracted extensive attentions and achieved many important applications in coatings, adhesives, plastics and biomedicine fields [9].

The physical properties of latex polymer film, such as Young’s modulus, tensile strength, elongation at break and solvent resistance, are very important criteria to determine its practical applications. These physical properties are closely depending on the macromolecular structure, the molecular weight, molecular inter- and intra- chemical crosslinking network structure, the size and morphology of the latex particles [10]. The polyacrylate-based latexes are kinds of industrious latexes being widely used due to their excellent anti-oxidation, oil resistance properties. However, compared to the solvent based polymers, their physical properties including film’s toughness, light fullness, water and solvent resistances are relatively weaker, and because of this, their practical utilizations are limited. In order to improve the actual performance of acrylate-based latexes, various kinds of techniques including soap-free emulsion polymerization, latex particle design with core-shell morphology and chemical modifications with silicon and fluorine related compounds are intensively developed. Particularly, there were not only a lot of research works concerning the syntheses and characterizations of the latexes with core-shell structures, but also there exist many reports related to the blends of water soluble epoxy resin with acrylate based latexes [11–17]. In these studies, special functional groups such as epoxy, N-methoxy-acrylamide, hydroxyl and carboxyl groups were first chemically incorporated into the main chain of acrylic polymers that can provide reactive sites for crosslinking. And then, for functional latexes with hydroxyl groups, the amino resins or blocked isocyanates were often used as crosslinkers and for latexes with carboxyl groups, the polycarbodiimide, polyaziridine and polyoxazoline were usually used as crosslinkers [18–20]. It has been noticed that, at present, chemically reactive acrylate-based latexes containing a special core-shell structure cross-linked by waterborne melamine-formaldehyde resin are rarely reported [21, 22]. Therefore, in this work, a core-shell composite polymer latex, specially, with P(MMA-*co*-BA) as the core, and P(EHA-*co*-GMA) as the shell was synthesized. Stable reactive latex blends were prepared by adding the latex with waterborne melamine-formaldehyde resin (MF) or urea-formaldehyde resin (UF). It was found that MF resin and UF resin can indeed act as an effective crosslinker via reaction with the reactive groups in the shell of the latex particles. Several physical properties of the polymer films cast from the latex blends are marked improved under higher thermal treatment temperature. The paper also analyses the detailed network structure formed via the crosslinking reaction and its possible molecular interaction mechanism.

## 2. Results and Discussion

**Figure 1.**
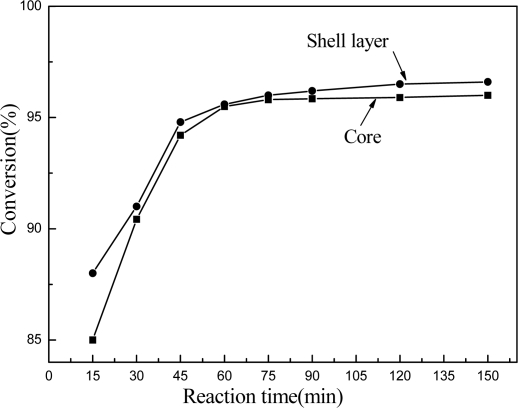
Time evolution of monomers conversion during the core and shell polymerization stages.

The chemical reactivity of latex during film formation can be affected greatly by the distribution of reactive groups in latex particles. Core-shell latex particles with reactive groups in the shell layer often provide latex with better reactivity. Two stages seeded semi-continuous emulsion polymerization were employed for constructing a core-shell morphology with hydroxyl and epoxy groups in the shell. A seeded latex was first prepared using special formulated monomers and surfactants as the core component, and then, the shell component consisting of designed monomers and other surfactants was added to the reaction system to produce the composite latex. In the polymerization stage of shell layer, reactive groups were introduced in the copolymer chains and crowded on the particle surface. In order to keep the stability of the polymerization, sodium bisulfite redox initiator system was used, and as a result, the emulsion polymerization can be carried out at a lower temperature of 60 °C. Figure 1 shows the monomer conversion curves of the core and shell polymerization steps. It was found that the monomer feeding time should be lasted 40 minutes for core layer and 60 minutes for shell layer, respectively. For each emulsion polymerization step, the monomer conversion can reach over 96% in 60 minutes after completing the monomer addition. It was also found that prolonging the reaction time couldn’t significantly raise the monomer conversion. Therefore, it is reasonable to conclude that the polymerization can be accomplished in 60 minutes after finishing monomer addition.

**Figure 2.**
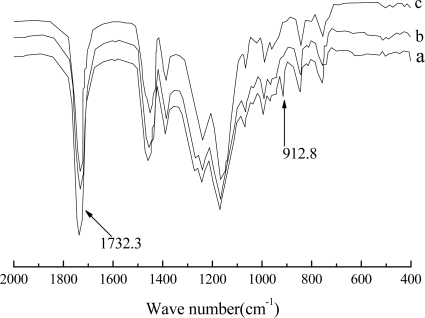
FTIR spectra of films cast from pure latex (a), AMF (b), and AUF (c) blends.

The macromolecular structure of copolymers synthesized was characterized by FTIR spectroscopy. Typical FTIR spectra of the pure latex and latex blends are shown in Figure 2. Figure 2 (a) presents the spectrum of the pure core-shell latex. As can be seen from Figure 2, the band at 1732.3 cm^−1^ is the absorption of C=O groups in acrylate polymer. In addition, the presence of epoxy groups is evidenced by the appearance of an absorption peak at 912.8 cm^−1^, which indicates that GMA segments have been successfully incorporated into the copolymer’s main chain via the second polymerization step.

**Figure 3.**
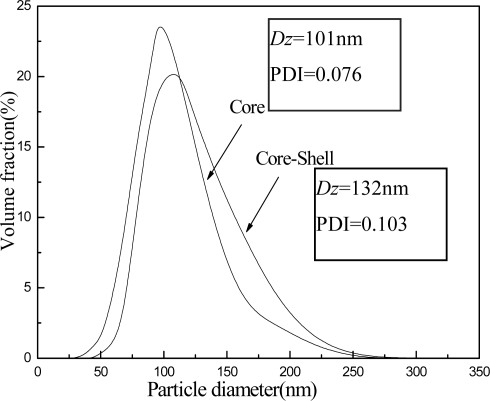
Particle sizes and distributions for the core component and core-shell latex.

Figure 3 presents the z-average diameter (*D_z_*), polydisperse index (PDI) and their particle size distribution curves of the core and complete core-shell particles. From Figure 3, we know that *D_z_* of the core particle is about 101 nm and shows a narrow distribution. On the other hand, *D_z_* of the core-shell particle is about 132 nm, and the increasing of PDI indicates that its distribution is relatively wide.

**Figure 4.**
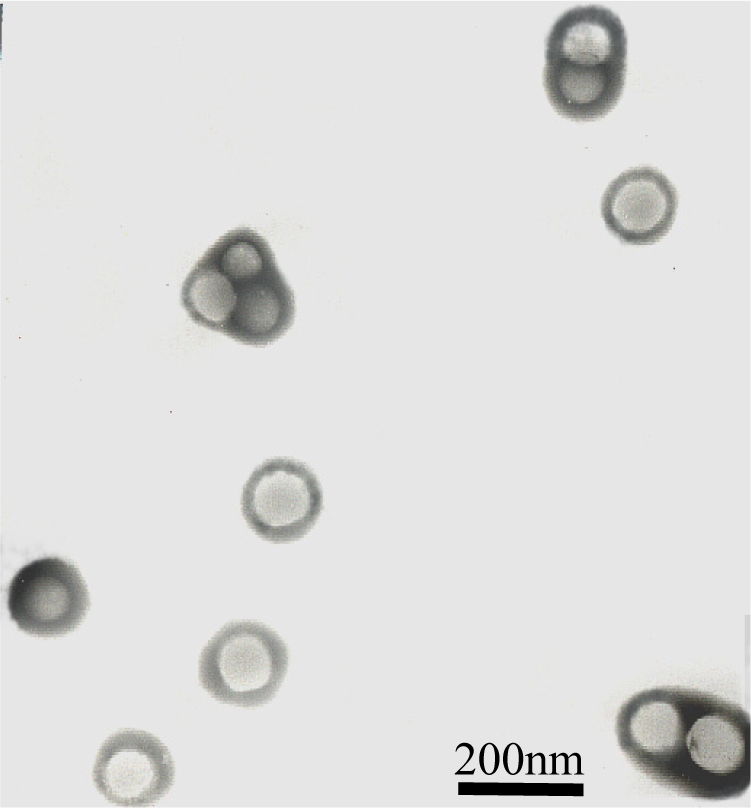
TEM Photo of the core-shell latex particles.

The core-shell structure of the latex particles can also be confirmed by a TEM photo in Figure 4. From Figure 4, the morphology of latex particles clearly demonstrates a desired micro-phase separated core-shell structure. The width of the shell layer is about 17 nm, which is consistent with the results of DLS measurement. And this suggests that the goal of our research for “particle design” is indeed fulfilled.

Due to the incorporation of chemically reactive groups into copolymer’s main chain, the crosslinking reactions between latex particles and waterborne MF or UF resin in the continuous phase of the latex blends may inevitably take place during film formation process under higher temperature. In order to understand the crosslinking mechanism and their physical properties affected by the addition of MF or UF resin, films of latex blends were heated at different temperature, and their structure and properties were studied. Figure 2(b) and Figure 2(c) show FTIR spectra of film AMF (blend of latex with MF resin) and AUF (blend of latex with UF resin) which were heated at 150 °C for 10 min, respectively. It was found that the absorption band of epoxy groups at 912.8 cm^−1^ disappears indicating a relatively complete chemical crosslinking reaction between two components.

Table 1 shows the effect of thermal treatment on T_g_ of polymer films. It was found that from Table 1, the glass transition temperatures for both pure latex and its blends, AMF and AUF, increase with the heating temperature. This indicates that the increase in the temperature of thermal treatment may actually increase the crosslinking densities for both pure copolymer and its blends, in turn, effectively raises their T_g_s.

**Figure 5.**
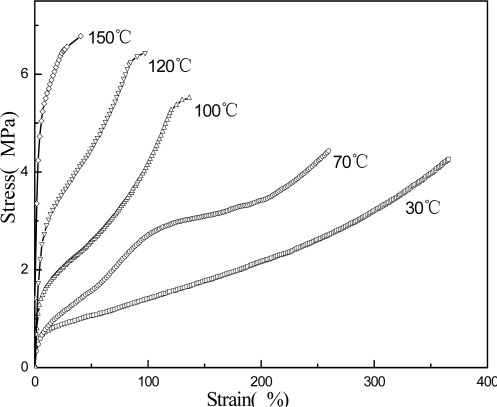
Stress-strain curves for films of pure latex.

**Figure 6.**
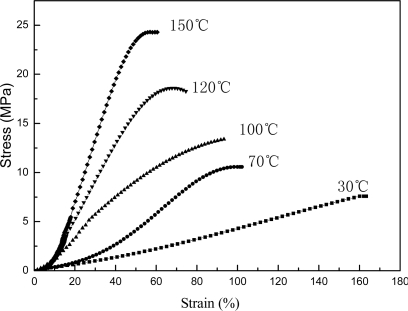
Stress-strain curves for films of AMF blend.

**Figure 7.**
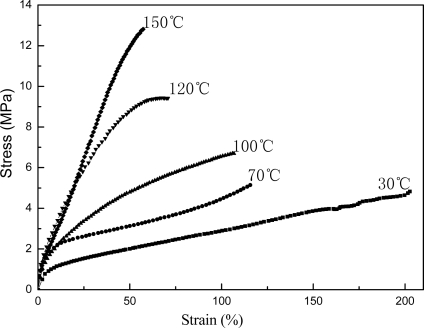
Stress-strain curves for films of AUF blend.

Figure 5, 6 and 7 present the mechanical properties for copolymer’s films cast from pure latex, AMF and AUF blends, respectively. It was found that the tensile strengths for films of pure latex and its blends increase following the increase in the thermal treatment temperature. However, their elongations at break decrease accordingly. On the other hand, the tensile strength for films of AMF and AUF present much higher values compared with the film of pure latex. The results may reveal that chemical reactions in the pure latex occured among the epoxy and hydroxyl groups, which results in formation of chemical crosslinking under the raising of the temperature. Furthermore, much more emical reactions in AMF and AUF blends may also be initiated among the epoxy and hydroxyl groups, and also, the amine and hydroxyl groups between latex particles and waterborne resins. As a result, it leads to a significant increase in the corsslinking densities of AMF and AUF films, and at the same time, enhances their crosslinking networkes.

**Figure 8.**
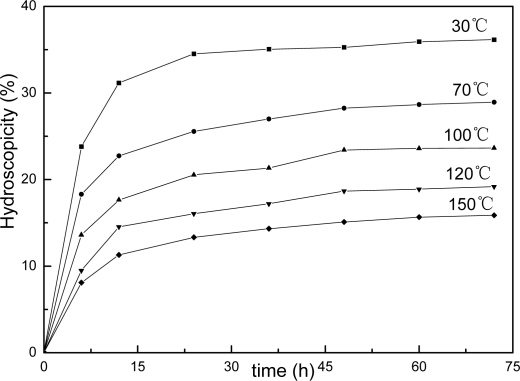
Hydroscopicity curves for films of pure latex.

**Figure 9.**
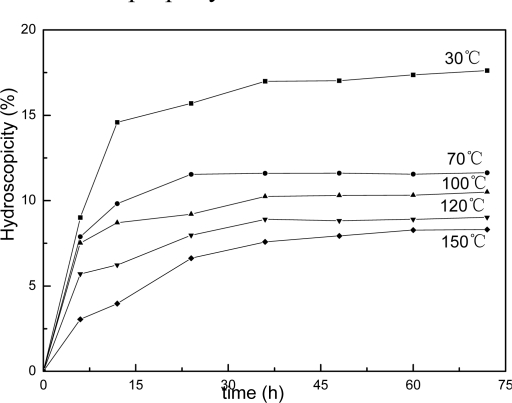
Hydroscopicity behavior for films of AMF blend.

**Figure 10.**
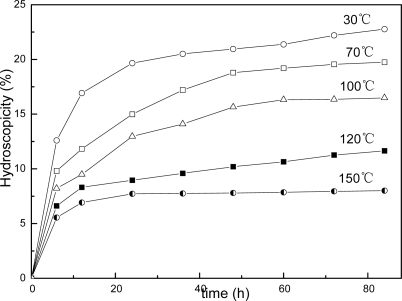
Hydroscopicity behavior for films of AUF blend.

This structure difference can also be reflected in copolymer’s hydroscopicity property as showing in Figure 8, 9 and 10. It is clear that the samples’ hydroscopicities decrease following increasing the thermal treatment temperature, however, the hydroscopicities for filmes of AUF and AMF blends present much lower values compared to the pure latex treated at the same temperature. The result still reveals a factor related to macromolecular network structure in polymer films. In general, the film formation process of polymer latex can be described in three stages [23]. First, water evaporates first from the surface of the latex, then the spherical particles move closer to each other and are deformed into hexagons, the interfacial tension between polymer and water/air causes gradual coalescence, at last, the observable boundaries among particles are deformed and disappear, the polymer chains of the particle surface interdiffuse each other, and a continuous film is finally formed. For AUF and AMF blends, certain amounts of MF or UF resin may deposit toward the interface of particles with the evaporation of water during film formation. These resin molecules can act as crosslinkers to link the latex particles with chemical bonds under higher temperature. The process can produce higher crosslinking densities in polymer films. The mechanical properties of films increase, and the film’s hydroscopicities decrease. Therefore, it is understandable that the reduction of hydroscopicities for films of AUF and AMF blends reflects actually the fact that their higher crosslinking densities and relative perfect network structures can effectively prevent water from penetrating into polymer’s matrix and enhance their water resistant property.

**Figure 11.**
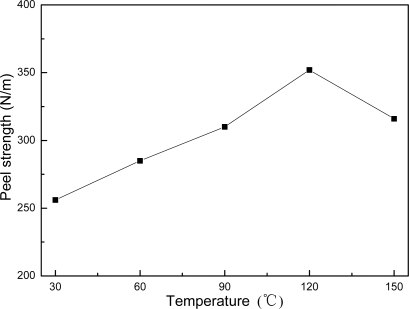
Relationship between the peel strength and thermal treatment temperature for the core-shell latex film.

It was also found that pure core-shell latex presents good adhesion strength onto a glass surface. The peel strength increases with the increase in thermal treatment temperature up to about 120 °C and then decreases with the further increase of the temperature as shown in Figure 11. The result may imply that the chemically reactive groups in the shell layer of core-shell latex particles can not only react among inter- and intra- macromolecules but also react with certain hydroxyl groups on the surface of a glass plate. Interestingly, AMF and AUF blends show little adhesion property even at a higher thermal treatment temperature. For AMF and AUF blends, most of the reactive groups on the surface of latex particles may quickly react with MF or UF resin, and there is little residual reactive groups in the film. As a result, the amount of chemical bonds between the film and glass plate were greatly reduced which decreases the adhesion strength.

## 3. Experimental Section

### 3.1. Materials

Methyl methacrylate (MMA) and Butyl acrylate (BA) were purchased from Bodi Chemical Ltd. (Tianjin City, China). 2-Ethylhexyl acrylate (EHA) and glycidyl methacrylate (GMA) were supplied as the industrial products by Hengguang Chemical Ltd. (Henan Province, China). The monomers, MMA, BA, EHA and GMA were all distilled before using. Sodium dodecyl diphenyl ether disulfonate (SDED) was purchased from Dalian Research & Design Institute of Chemical Industry (Liaoning Province, China), and used after receiving without further distillation. Polyoxyethylene nonyl ether (CO-897) and 1-allyloxy-2-hydroxy propane sulfonate sodium (AOPS-1) were purchased from Xi’an Tianyun Ltd. (Xi’an City, Shannxi Province, China), and used without further purification. Melamine formaldehyde resin (MF, 55–60% solid content, 15–27 DIN 4 sec.), was supplied as the industrial product by Dynea Chemical Ltd (Shanghai City, China). Buffer solution (NaH_2_PO_4_-Na_2_HPO_4_, pH=7.0), waterborne melamine-formaldehyde resin (MF) and urea-formaldehyde resin (UF) were obtained from Dynea Chemical Ltd (Shanghai City, China).

### 3.2. Synthesis of core-shell latex particles

The latex particles with a core-shell structure were prepared via a seeded semicontinuous emulsion polymerization. All the experiments were carried out in a 250 mL four-necked flask equipped with an overhead stirrer, a thermometer, a nitrogen inlet tube and a feed inlet tube. The detailed recipes used were listed in Table 2. Specifically, 10g of distilled water was first charged into the flask, and the system was purged with nitrogen gas at 60 °C for 15 min, then 10g of pre-emulsified monomers used as the core component together with small amount of initiator was added to the flask. 5 min late, the remaining core component was added dropwise to the reactor within 40 min. The system was kept at 60 °C for additional 1 h to allow it conducting polymerization. Then, the pre-emulsified monomer using as the shell component was slowly added to the reactor within 1 h. After completing the addition, the system was continuously maintained polymerization at 60 °C for 3 h. All polymerization were carried out under a nitrogen atmosphere.

### 3.3. Preparation of latex blend

100 g of latex synthesized was charged into a 250 ml flask stirring vigorously at room temperature. At the same time, certain amount of MF (the mass ratio, latex/MF = 100/1) or UF (the mass ratio, latex/UF = 60/1) water solution (5 wt%) was added together with small amount of p-toluene sulfonic acid as the catalyst to the system, respectively. After stirring for about 30 minutes, the mixture showed uniform and homogeneous indicating that the stable AMF (blend of latex with waterborne MF) or AUF (blend of latex and waterborne UF) blend was obtained.

### 3.4. Measurement and Characterization

#### Measurement of the monomer conversion

The monomer conversions were determined gravimetrically. Samples (*m*_0_) withdrawn from the reactor during the polymerization were short-stopped with a solution of 1% hydroquinone in water. And then the sample was dried to constant weight (*m*_1_) at 110 °C. The solid content of the system was evaluated by the following equation.

$$s=\frac{m_{1}}{m_{0}}\times 100\%$$

The monomer conversion in the core stage was evaluated by the following equation.

$$\alpha_{\left(\text{core}\right)}=\frac{m_{l}\times s-m_{u}}{m_{c}}\times 100\%$$

where *m_l_*, *m_u_* and *m_c_* were the weight of emulsion in reactor, non-volatile residue and total monomer of core component, respectively.

The conversion in the shell stage was evaluated by following equation.

$$\alpha_{\left(\text{shell}\right)}=\frac{m_{l}\times s-m_{c}\times \alpha_{\left(\text{core}\right)}-m_{u}}{m_{s}}\times 100\%$$

where *m_s_* was the weight of total monomer of shell component.

#### Measurement of Particle size and its distribution

Particle size and its distribution of latexes were measured by dynamic light scattering (DLS, Malvern Zetasizer Nano-ZS). Samples were diluted to low concentrations (<10^−3^ g/L) and then deposited for the DLS analysis. The mean particle size was characterized by z-average diameter (*D_z_*), and the particle size distribution was characterized by polydispersity index (PDI) together with the curve of particle size distribution.

#### Particle Morphology

Taking a drop of latex to deposit it on a copper net and allowing it to set for 5 minutes. The sample prepared was dyed with phosphototungstic acid for 3 minutes, and dried at room temperature. The particle morphology was inspected on a Hitach-600 Transmission Electron Microscopy (Japan) at 80 KV.

#### Tensile Strength Measurement

The tensile strength of films cast from latex blends was measured using a CMT 6000 Electromechanical Testing Machine (Sans Co., Shenzhen, China) with a crosshead speed of 60 mm/min at room temperature. Sample size was 80 mm×6 mm×1 mm. The tensile strength values reported herein were the average of 6 samples.

#### DSC Analysis

Differential scanning calorimetry (DSC) studies were conducted on a TA MDSC 2910 instrument under a dry nitrogen atmosphere at constant heating rate of 10 °C/min in a temperature range −50 °C to 100 °C.

#### Hydroscopicity of Polymer Films

The hydroscopicity of copolymer’s films was tested according to ASTMD 570–8. The weighted latex films were dipped in distilled water at 25 °C for 48 hours. Then, the free water on surface of films was cleared by filter paper, and the film was weighted again. The water absorption ratio of films was calculated by following equation: Water Absorption Ratio (Wt %) = (W_1_ – W_0_)/W_0_×100%, where W_0_ is the weight of dry film, and W1 is the weight of the film absorbing water, respectively.

#### Peel Strength Measurement

Peel strength was measured at room temperature using an EMT2000 Electromechanical Testing Machine (Sans Co., Shenzhen, China) with a crosshead speed of 100 mm/min. The sample size for testing was 30 mm×150 mm×5 mm. The samples were first placed in contact under a pressure of 0.8 MPa for 10 s so as to achieve desired adhesion strength. The values obtained were the average of five samples tested.

## 4. Conclusions

By using two steps seeded semi-continuous emulsion polymerization, polyacrylate based composite latexes with a core-shell morphology can be synthesized under the special formulated recipes at 60 °C. Stable reactive latex blends were prepared by adding the latex with waterborne melamine-formaldehyde resin (MF) or urea-formaldehyde resin (UF). The mechanical property, water resistance and glass transition temperature of the polymer films cast from the latex and its blends can be marked improved under higher thermal treatment temperature. The physical properties are subjected to the adjustment of the thermal treatment temperature due to the chemical reaction via the reactive groups incorporated for polymer and its main chain. The higher the thermal treatment temperature, the better physical properties for copolymers are achieved.

## Figures and Tables

**Table 1.** Effect of thermal treatment temperature on T_g_s of polymer films.

Thermal treatment temperature / °C	30	70	100	120	150
T_g_ (Pure latex) / °C	18.2	19.11	19.17	20.74	26.11
T_g_ (AMF blend) / °C	18.89	19.22	20.89	23.42	29.58
T_g_ (AUF blend) / °C	19.10	19.25	20.58	22.81	28.19

**Table 2.** The recipes of copolymer for core-shell emulsion polymerization.

Component	Core component (g)	Shell component (g)
CO-897	0.4	0.3
SDED	0.3	0.2
AOPS-I	0.10	–
MMA	10	–
BA	10	–
GMA	–	2.5
EHA	–	7.5
Buffer solution (pH=7.0)	5	–
(NH_4_)_2_S_2_O_8_	0.15	0.10
NaHSO_3_	0.05	0.03
H_2_^O^	20	10
